# Neurophysiological modulation of rapid emotional face processing is associated with impulsivity traits

**DOI:** 10.1186/s12868-015-0223-x

**Published:** 2015-12-09

**Authors:** Takahiro Soshi, Takamasa Noda, Kumiko Ando, Kanako Nakazawa, Hideki Tsumura, Takayuki Okada

**Affiliations:** Department of Forensic Psychiatry, National Center of Neurology and Psychiatry, National Institute of Mental Health, 4-1-1, Ogawahigashi, Kodaira, Tokyo 187-8553 Japan; Department of Psychiatry, National Center Hospital, National Center of Neurology and Psychiatry, 4-1-1, Ogawahigashi, Kodaira, Tokyo 187-8551 Japan

**Keywords:** Emotional face processing, Visual oddball paradigm, Electroencephalogram, Event-related potential, Visual mismatch negativity, Impulsivity

## Abstract

**Background:**

Sensori-perceptual processing of emotional stimuli under attentive conditions effectively prevents response disinhibition. This is observed saliently in low-impulsive people, because of their high sensitivity to warning signals, such as emotional faces. Results from human neurophysiological studies have been used to develop a dual detector model for early sensori-perceptual processing. A transient detector mechanism is related to automatic neurophysiological arousal in response to warning signals, which is reflected by early frontal event-related potential effects. The memory-based detector mechanism is associated with subsequent mismatch negativity (MMN), which reflects a short-term memory trace of signals. Based on previous findings, we predicted that impulsivity affects functional associations among the dual detector mechanisms, and modulates early frontal and/or MMN activities. In the present study, we recorded electroencephalograms for twenty-one healthy adults using a visual oddball paradigm with neutral faces as frequent stimuli, and angry and happy faces as infrequent stimuli. We measured the impulsivity traits by a self-report scale (the Barratt Impulsiveness Scale, 11th version).

**Results:**

Main findings were that only happy faces increased early frontal negativity and subsequent occipital visual MMN (vMMN) for emotional change, and these neurophysiological effects positively correlated with each other in a temporally causal manner. However, an impulsivity sub-trait positively correlated selectively with vMMN for the happy faces.

**Conclusion:**

These findings demonstrate that higher impulsivity is associated with attenuated vMMN for emotional change detection in healthy populations, potentially because of weakened fronto-occipital functional connection that is responsible for the dual detector mechanism.

**Electronic supplementary material:**

The online version of this article (doi:10.1186/s12868-015-0223-x) contains supplementary material, which is available to authorized users.

## Background

Observing emotional expressions in other people frequently evokes involuntary responses. In socially favorable situations, happy faces frequently evoke positive emotions in surrounding people and promote interpersonal interaction [1]. However, even emotionally positive faces, if they are mismatched to the circumstances, sometimes evoke disinhibited impulsive responses that can lead to tragic consequences. While emotional expressions automatically affect sensori-perceptual processing [2–4], sensori-perceptual processing of emotional stimuli under attention may prevent response disinhibition [5, 6]. On the other hand, sensori-perceptual processing under attention may depend on sensitivity to salient stimuli, such as emotional faces as a function of personality [7], and be associated with prefrontal-sensory functional connectivity responsible for top-down processing [8]. Therefore, it might be beneficial to investigate neuropsychological modulation in sensori-perceptual processing of alert signals to develop coping strategies to suppress impulsive responses.

Oddball paradigms have been frequently used to investigate neural foundations for preattentive detection of stimulus change in various sensory modalities, such as visual [9–12], somatosensory [13, 14], olfactory [15, 16], and auditory [17, 18] systems. In the most-widely studied auditory paradigms, frequent and infrequent auditory stimuli are randomly presented. Infrequent stimuli, compared to frequent stimuli, mainly increase an event-related potential (ERP) called mismatch negativity (MMN) for stimulus change that appears around 200 ms after stimulus onset [17, 18]. Because MMN can be observed in preattentive situations [19, 20] and across several sensory modalities, it is considered a neurophysiological marker of automatic sensori-perceptual change detection of stimuli [21].

Previous studies have argued that neural activities for stimulus change detection are based on a dual detector mechanism [22–24]. One is a transient detector mechanism, which automatically evokes an arousal response and facilitates a motor response [23, 25]. The arousal response is generally related to alertness [7], which is evoked by warning signals. This mechanism updates neuronal refractoriness to frequent stimuli by warning signals [24]. In addition, the mechanism may be related to a basic neurophysiological process, for example, early cortical activities or N100 (N1) appearing around 100 ms post-stimulus [22, 26].

The second is a stimulus-change detector mechanism [23, 27], which is associated with a short-term sensory memory trace of stimuli [19]. This detector mechanism is reflected by MMN, which is distinguished from frontal ERP effects of an involuntary attentional shift after stimulus change detection [25, 28]. Taken together, sensori-perceptual change detection is based on the temporally ordered neural activities for automatic arousal and subsequent detection of stimulus change.

The present study investigated the neuropsychological factors affecting neural activities for sensori-perceptual processing of emotional stimuli. Previous studies suggest that impulsivity traits affect the dual detector mechanisms [29–31]. Furthermore, impulsivity traits are considered a complex behavioral trait, difficult to comprehend [32], and are related to various behavioral patterns, such as a lack of concentration or self-control, cognitive instability, disinhibition, non-perseverance, lack of future planning, sensation seeking, and risk taking (see for review, [33]). A number of personality theories have defined impulsivity multifariously as “a sub-trait of extraversion which is a basic dimension of personality,” “a basic temperament distinguished from extraversion,” or one of the major personalities, “sensation seeking” [34]. More recently, impulsivity has been conceptualized as “a predisposition toward rapid, unplanned reactions to internal or external stimuli without regard to the negative consequences of these reactions to the impulsive individual or to others” [35].

To define the association between impulsivity and the dual detector mechanisms, several theories have been proposed [31], including the general arousal theory [30] and the short-term information transfer/short-term memory theory [7]. These models indicate that impulsivity traits are differently associated with sensitivity to alert signals. That is, low-impulsive people perform better than high-impulsive people in simple perceptual tasks, because higher sensitivity to alert signals suppresses performance decrease in lower impulsive people [31, 36]. This is further supported by neurophysiological observations [34]: low-impulsive people attenuate early-evoked potentials as a function of stimulus intensity [37], because they likely possess an optimal level of sensitivity to alert stimuli. Taken together, these data suggest that impulsivity influences the arousal response and the subsequent sensori-perceptual change detection because a functional connection exists between the two detector mechanisms.

To examine how impulsivity traits affect the dual detector mechanisms, we used a visual oddball paradigm [9]. Similar to auditory MMN, visual MMN (vMMN) is elicited to stimulus change even without direct attention. vMMN effects are observed primarily in posterior occipital sites during the intervals from about 100 to 300 ms after stimulus onset [10, 38]. Among various types of visual stimuli (see for the recent review, [24]), socially meaningful face stimuli have also been used [4, 10, 11, 39–43]. Facial pictures [11] and line drawings [43] induce comparable vMMN effects. Change of face orientations can also elicit vMMN [12]. Thus, vMMN is associated with stimulus- or feature-change detection, which updates a transient memory representation of preceding stimuli with a time span shorter than 300 ms [44, 45].

The present study utilized a short stimulus duration of 200 ms and stimulus-onset-asynchrony of 500 ms, which is adequately short to construct a short-term memory representation of frequent stimuli. Neutral face pictures were used as frequent stimuli and angry and happy faces from the same person were used as infrequent stimuli to investigate neurophysiological correlates of emotional change detection. White circles were used as a distracting target for a button response to prevent participants from paying direct attention to emotional expressions [4]. Electroencephalogram (EEG) was recorded while participants (21 healthy adults) were performing the task. To evaluate frontal and occipital neurophysiological effects for emotional change, we separated the activities localized in these two areas by the independent component analysis (ICA) [46]. Emotional change effects were examined by comparisons of the amplitudes between infrequent and frequent faces. Temporally constrained functional connections between early frontal and subsequent vMMN effects were also examined for top-down emotional face processing, because impulsivity may affect frontal neural activities crucial for top-down processing [47]. Finally, neurophysiological effects were correlated with impulsivity traits, behavioral performances, and emotional assessments of faces.

It remains ambiguous whether positive or negative emotional bias appears more saliently in neurophysiological response for emotional face change and neurobehavioral correlations with impulsivity, because both emotional effects have been reported earlier [10]. However, based on the dual detector model, it is anticipated that early frontal activities generally enhance subsequent vMMN effects. It is also predicted that impulsivity is related to changes in early frontal effects and occipital vMMN for emotional change. This implies that people with higher impulsivity may exhibit lower sensitivity and more rapid habituation to emotional change [34], and hence, likely more attenuate early frontal activities and subsequent vMMN. On the other hand, if impulsivity also affects fronto-occipital functional connectivity because it attenuates top-down processing, differences in impulsivity-related correlations will be visible between early frontal and vMMN activities.

## Methods

### Participants

Twenty-one healthy Japanese adults participated in the experiment during the daytime (10 a.m. to 3 p.m.). Their socio-demographic profiles are summarized in Table 1. Mean ages and education levels of the male and the female participants were not significantly different (Mann–Whitney: age, *U* = 41.500, *p* = 0.783; education: *U* = 36.500, *p* = 0.503). Mental health of participants was assessed according to SCID-I/NP (Structured Clinical Interview for DSM-IV-TR Axis I Disorders, Non-patient Edition) [48] by an experienced psychiatrist or a clinical psychologist. Exclusion criteria included historical or existing psychiatric illness, brain injury, cognitive impairment, substance abuse, and inability to understand Japanese language. Right-handedness was assessed using the Edinburgh handedness inventory [49]. All participants had normal or corrected to normal vision. The present study was conducted in accordance with Declaration of Helsinki. All participants provided written informed consent prior to the experiment, based on the research protocol approved by the Ethical Committee of the National Center of Neurology and Psychiatry (NCNP).

**Table 1:** Demographic profiles and impulsive traits of the healthy participants (*n* = 21)

	Female (*n* = 15)		Male (*n* = 6)	
	Mean	SD	Mean	SD
Age (years)	29.7	9.9	28.2	10.1
Education (years)	15.7	2.5	17.7	4.3
BIS-11				
AI	14.3	2.8	14.2	2.9
MI	20.5	2.2	24.7	4.8
NPI	24.9	3.6	26.8	2.0

*SD* standard deviation, *BIS-11* the Barratt Impulsiveness Scale, 11th version, *AI* attentional impulsivity, *MI* motor impulsivity, *NPI* non-planning impulsivity

### Visual oddball paradigm

**Fig. 1:**
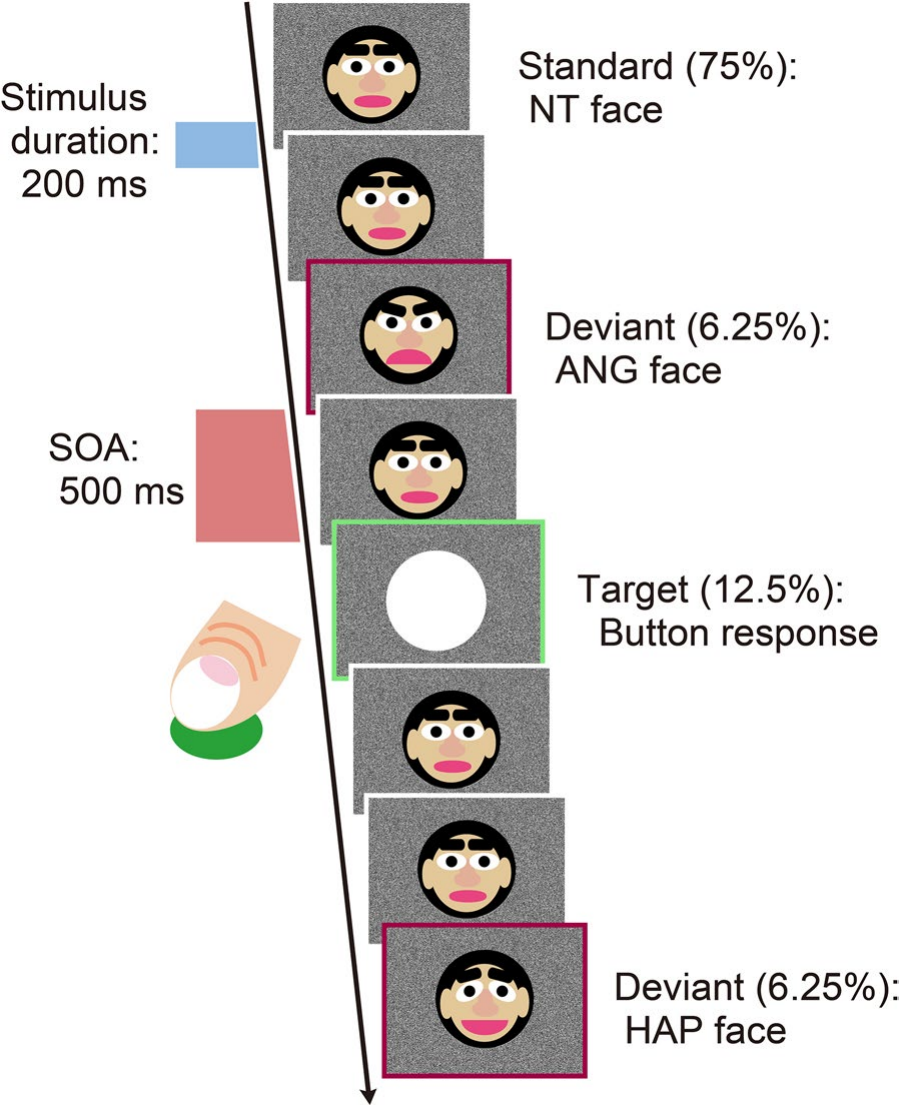
Visual oddball paradigm. The paradigm included the frequent neutral (NT; 75 %), and infrequent angry (ANG; 6.25 %) and happy (HAP; 6.25 %) *gray-scaled faces*, which were represented by self-making illustrations, and white circle targets (12.5 %). These stimuli were pseudo-randomly presented in the center of random-dot backgrounds so that the ANG and HAP faces or targets did not appear successively in each of the three blocks (160 stimuli for each block). Participants were instructed to press a *button* with their right index finger as rapidly as possible, when targets appeared. All stimuli were presented for 200 ms with the stimulus-onset-asynchrony (SOA) fixed at 500 ms. Response times for targets appearing immediately after the ANG and HAP faces were averaged to examine interference effects on button responses. Averaged event-related potential (ERP) waveforms were produced separately for the NT, ANG, and HAP faces

The participants sat on a chair inside a sound attenuated chamber (about 38 dB sound pressure level and 70 lux around the experimental desk surface) and faced a 19-inch monitor placed 0.9 m in front of their heads. They performed a visual oddball task (Fig. 1). In the present paradigm, frequent neutral (NT: 120 stimuli; 75 %), infrequent angry (ANG), and happy (HAP) faces (10 stimuli × 2 conditions = 20 stimuli; 12.5 %), and white circle targets (20 stimuli; 12.5 %) were pseudo-randomly presented in each of three blocks (160 stimuli × 3 blocks = 480 stimuli). Infrequent faces did not appear successively and were preceded by at least two NT faces (2–7 stimuli). Mean numbers of preceding NT faces were not significantly different between the ANG and HAP conditions (ANG: 3.7 ± 1.2 faces; HAP: 3.7 ± 0.9 faces; *t*_(54)_ = 0.059, *p* = 0.953). Ten NT faces always appeared at the beginning of each block. Each face stimulus appeared in the center of the display for 200 ms, and the stimulus-onset-asynchrony was fixed to 500 ms (MTS0410, Medical Try System, Tokyo, Japan). The visual angles of the stimuli were 10.285° vertically and 9.211° horizontally. Presentation orders of the three blocks were counterbalanced across the participants. Frequent stimuli consisted of female and male faces, 30 each, which were presented twice in each block. ANG and HAP faces were obtained from the same females, and were divided into three sets (10 stimuli for each emotional expression) with no repetition within a block. The present study differentiated emotional expressions of the same persons between frequent and infrequent faces to examine emotional change detection [10]. Participants were instructed to press the button on the response pad as rapidly and correctly as possible using their right index fingers, when the target was presented. They were not given information in advance that the stimuli included three types of emotional faces. After the experiment, participants were asked what they noticed about stimuli during the task. All participants reported that they did not direct attention to stimulus change during performing the task. Participants assessed emotional expressions of randomly selected 30 faces of three emotional expressions (10 faces for each emotional expression) by a ten point Likert scale (10 = ”happy”; 1 = ”angry”) after completion of the experiment. Emotional distances between the ANG or HAP and NT faces were defined as absolute difference scores (|ANG or HAP minus NT|).

### Experimental stimuli

Face stimuli were collected from the Karolinska Directed Emotional Faces (KDEF: http://www.emotionlab.se/resources/kdef) [50] and the NimStim database (http://www.macbrain.org/resources.htm). Females and males faces, fifteen each, were selected from each database, for a total of 60 individuals (30 faces × 2 databases) with NT, ANG, and HAP emotional expressions for each. All stimuli were converted into gray-scale, levels of brightness were equated based on the mean score of 112 (0–255) for a reversed triangle area (40,681 pixels) covering the main facial part with eyes, nose, and mouth, and were transformed into a circular form (a diameter of 372 pixels). White circle targets had the same size as face stimuli. Moving random dot screens (1158 × 872 pixels) were used as the stimulus background to attenuate afterimages of stimuli. A black fixation cross constantly appeared in the center of the display without face stimuli.

### Measurement of impulsivity traits

Impulsivity trait was measured with the Barratt Impulsiveness Scale, 11th version (BIS-11) [51, 52]. The BIS-11 contains 30 items clustered into three types of the second order impulsivity, including attentional impulsivity (AI) [(8 items): 8–32 scores], motor impulsivity (MI) [(11 items): 11–44 scores], and non-planning impulsivity (NPI) [(10 items): 10–40 scores]. AI is composed of the first order impulsivity of attention (focusing on the task at hand) and cognitive instability (thought insertions and racing thoughts), and is characterized as difficulty in concentration. MI combines motor impulsiveness (acting on the spur of the moment) and perseverance (a consistent life style) and is related to actions without consideration. NPI consists of self-control (planning and thinking carefully) and cognitive complexity (enjoy challenging mental tasks), and is defined as a lack of future planning [51]. The BIS-11 is scored using a four point Likert scale (4 = very true for me; 3 = somewhat true for me; 2 = somewhat false for me; 1 = very false for me). Greater scores indicate higher impulsivity traits self-evaluated.

### Electroencephalogram recording and analyses

EEG data for individual trials were recorded (1000 ms before and after stimulus onset) from the four midline and two bilateral occipital Ag–AgCl scalp electrodes (Fz, Cz, Pz, Oz, O1, and O2; *φ* = 10 mm) with a commercialized bio-amplifier system (MEB-2300, NIHON KODEN Corp., Tokyo, Japan). Additional three electrodes were attached around the eyes to monitor horizontal electro-oculogram (HEOG: left-upper minus right-upper) and vertical EOG (VEOG: left-upper minus left-lower). All electrodes were on-line referenced to the linked mastoids. The ground electrode was positioned on a participant’s chin. EEGs were recorded at a sampling rate of 1024 Hz with a band-pass frequency ranging from 0.1 to 100 Hz. The impedance was set below 5 kΩ.

Stored EEGs (−1000 to 1000 ms) were filtered with a band pass frequency ranging from 0.5 to 40 Hz. VEOG components were removed from individual EEGs by a regression method [53]. EEGs were linearly regressed by individual EOGs, and regression coefficients (*β*) were calculated (*mEEG*_*i*_ = *β*_*i*_ × *VEOG*_*i*_ + *C*_*i*_; *mEEG* = measured EEG; *C* = the *y*-intercept of the equation; *i* = the number of EEG epochs). VEOG-free EEGs were calculated by the subtraction equation (*estEEG*_*i*_ = *mEEG*_*i*_ − *β*_*i*_ × *VEOG*_*i*_; *estEEG* = estimated EEG). After removing VEOGs, EEG epochs from 100 ms before until 500 ms after the onset of faces were spliced out separately for the NT (120 epochs × 3 blocks), ANG (10 epochs × 3 blocks), and HAP (10 epochs × 3 blocks) faces. Individual averaged waveforms were calculated after baseline correction (mean potentials during the baseline interval from −100 to 0 ms) and artifact rejection of residual artifacts, such as drifts (peak-to-peak amplitudes of ±75 μV). Rejection rates were zero in almost all of participants (NT: 0.56 ± 1.9 %; ANG: 0.63 ± 1.3 %; HAP: 0.79 ± 1.3 %). All EEG analysis steps mentioned above were carried out with customized MATLAB functions (the Mathworks, Tokyo, Japan).

The present study conducted the ICA with the FastICA algorithm [46] to separate frontal and occipital activities and to examine their functional connections for emotional change detection. ICA is a blind source separation method in which mixed signals (*x*) are observed in several locations, such as electrode positions are separated into non-Gaussian, statistically-independent components (*s*) with an upper limit of number of observed locations (*s* = *A*^−1^*x*; *A*^−1^ = an inversed matrix of a mixing matrix *A*). Although ICs estimated from surface potential data are not necessarily equal to unique sources of neuronal populations, it is effective to attenuate crosstalk of multiple signals and increase localization properties of relevant neural activities [54]. ICA in the present study aimed to estimate a mixing matrix *A* for the original data *x*, and to estimate ICs localizing in frontal and occipital surface areas. Individual average ERP data (615 time points × 6 electrodes) underwent centering, i.e. mean values were subtracted from the original data *x* to make *x* a zero-mean data. Centered data were converted into a normalized covariance matrix (6 × 6), which was decomposed into eigenvalues (*D*) and eigenvectors (*E*) (principal component analysis part). While preprocessing for ICA, using eigenvalues and eigenvectors, new data (_*wh*_*x*: whitened *x*) were obtained by whitening of the centered data *x*, so that latent components did not correlate with each other and their variances are equal unity $[_{wh} x = sqrt\left( D \right)^{ - 1} {\, \times\,} E^{\text{T}} {\,\times\,} {\it {x}};sqrt = {\text{ square root function}};E^{\text{T}} = {\text{ a transposed matrix of}} \, E]$. At this computational stage, numbers of dimensions or ICs with larger eigenvalues (>1) were specified for use in the present ICA (two dimensions in the present study). The FastICA algorithm is to estimate a weight vector *W* (that is, *A*^−1^), so that ${\it {W}}^{\text{T}} {}_{wh}{\it{x}}$ is provided a maximum non-Gaussianity (non-random, clearly clustered structure), which is represented by neg-entoropy $\left[ {J\left( y \right) \, = H\left( {y_{\text{gauss}} } \right) - H\left( y \right);y = W^{\text{T}} {}_{wh}{\it{x}};H\left( y \right) \, = {\text{ entropy of }}y} \right].$ The algorithm actually approximates a maximal neg-entropy so that ${\it {W}}^{\text{T}} {}_{wh}{\it {x}}$ possesses the least Gaussian distribution [46]. We adopted the one-by-one estimation approach (deflation) and the non-linear approximation method (pow3) in the present analysis. These functions are implemented in the MATLAB-based FastICA package, which is available from the website: http://research.ics.aalto.fi/ica/software.shtml.

### Statistical analyses

#### Emotional facial assessments

Assessment scores of the three facial expressions were compared with the Friedman test. When significant main effects were obtained, pair-wise comparisons were conducted with the Wilcoxon test. To examine differences in emotional distances (absolute difference scores between frequent and infrequent faces) between the ANG and HAP faces, emotional distances were directly compared with the Wilcoxon test. An α level of *p* < 0.05 was considered significant in this and all subsequent tests.

#### Response times for targets immediately after the appearance of infrequent emotional faces

To examine how the infrequent faces affect target responses immediately after their appearance, mean response times (RTs) were compared between conditions with a permutation *t* test [55]. This method is based on the conception that tested distributions of statistical values are empirically derived by multiple permutation analyses of collected samples to avoid a type I error in multiple analyses. Data for paired conditions were repeatedly re-sampled from the three emotional conditions across participants and conditions so that the same patterns of re-sampling were not included. Re-sampled data were compared with paired *t* tests to obtain dummy *t* values. Because the number of permutations is too vast to compute overall *t* values, permutation procedures were repeated 10,000 times. Original *t* values were tested by permutation distributions of 10,000 dummy *t* values. When original values were outside a 95 % confidence interval (CI), the values were considered significant at a corrected *α* level of *p* < 0.05. We reported both original *t* values and a 95 % CI of dummy *t* values. The RTs for the ANG and HAP faces were also linearly correlated with emotional assessment scores and impulsivity traits to examine the influence of emotional change and impulsivity traits on the target RTs. We also used a permutation procedure. Data *X* corresponded to RTs for targets after the ANG or HAP faces, and data *Y* to emotional assessment or impulsivity scores. First, data *Y* were scrambled across participants and items, and were repeatedly correlated with data *X* by the Pearson’s method. A total of 10,000 correlation coefficients were obtained for emotional or impulsivity scores in each face condition. Original coefficients were tested by permutation distributions of 10,000 dummy coefficients. When original coefficients were outside 95 % CIs, the coefficients were considered significant at an *α* level of *p* < 0.05, corrected. Permutation tests were conducted with customized MATLAB functions.

#### Fitting tests of frontal and occipital independent components

To confirm whether ICs reflect frontal or occipital activities, averaged waveforms at the six electrodes were linearly regressed by each IC. Regression coefficients (*β*) were calculated to examine fitting properties between the ICs and average ERP waveforms for each participant (average ERP_*i*_ = *β*_*i*_ × *IC*_*j*_ + *C*_*i*_; *i* = Fz, Cz, Pz, Oz, O1, O2; *j* = 1, 2; *C* = the *y*-intercept). More positive coefficients indicate better fitting. Coefficients for each IC in each type of emotional faces were compared between the frontal (Fz) and occipital (Oz) sites with the Wilcoxon test to examine localization properties of the ICs.

#### Neurophysiological effects for detection of emotional face change

We first compared average amplitudes of the frontal and occipital ICs between the NT and infrequent (ANG and HAP) faces. Based on visual inspection of difference waveforms (infrequent minus frequent), the frontal IC could be separated into three temporal phases. The first phase corresponded to the interval of early frontal negativity (EFN) effects, which comprised N1 (55–145 ms), before the second phase of middle frontal negativity (MFN; 145–205 ms) with a clear negative peak. The third phase corresponded to the interval of late frontal negativity (LFN; 205–500 ms) with a weak sustained effect after the convergence of the MFN. For the occipital IC, vMMN for the infrequent faces was observed in middle intervals (145–345 ms). These four temporal windows were compared between the frequent and infrequent faces also using permutation *t* tests. Data for the two conditions were repeatedly re-sampled from the four ERP intervals across participants and conditions, and were compared with paired *t* tests to obtain 10,000 dummy *t* values. When original values were outside a 95 % CI, the values were considered significant at an *α* level of *p* < 0.05, corrected.

Later, the infrequent condition was divided into ANG and HAP conditions and the three conditions were compared with a one-way analysis of variance (ANOVA) with the within-participants factor of emotion (NT, ANG, and HAP). The same intervals were used for the ANOVA. When a significant main effect was observed, pair-wise comparisons were performed with the Fisher’s least significant difference (LSD) method. When a significant trend (*p* < 0.1) was observed, planned pair-wise comparisons were performed in a similar manner to explore emotional change effects. The Greenhouse-Geisser correction was not performed in ANOVAs, because of no violation of sphericity.

#### Temporally causal connection between early frontal and occipital vMMN effects

Relational direction between EFN and vMMN is a unidirectional path from EFN and vMMN under the constraint of temporal order. Hence, we correlated EFN as a seed with occipital vMMNs for the ANG and HAP faces. The EFN interval (55–145 ms) was divided into nine intervals with a 10 ms step, and each interval was correlated with vMMN (145–345 ms) divided into twenty intervals. Temporally causal connection was tested by a permutation procedure. Data *X* corresponded to EFN amplitudes, and data *Y* to vMMN amplitudes. Data *Y* were scrambled across participants and were repeatedly correlated with data *X* by the Pearson’s method. A total of 10,000 correlation coefficients were obtained for each analysis. Original coefficients outside 95 % CIs were considered significant at an *α* level of *p* < 0.05, corrected. Chi-square tests with a 2 × 2 table (emotion × significance) were performed to examine proportional differences in number of significant intervals between the ANG and HAP faces. Significant intervals were merged and were reanalyzed with permutation procedures, and were represented graphically for easy reference.

#### Correlation between neurophysiological and behavioral measure

Neurobehavioral properties of vMMN and EFN were tested similarly by permutation correlation analyses, concerning behavioral measurements (RT, raw and different emotional assessment scores) and impulsivity traits (AI, MI, and NPI). Intervals of vMMN and EFN were also divided into sub-intervals with a 10 ms step, and were correlated with behavioral and impulsivity measures. The permutation also counted 10,000 times in a non-overlapping manner for each analysis, and original coefficients outside 95 % CIs were considered significant at an *α* level of *p* < 0.05, corrected. Chi square tests with a two-way table (emotion × significance) were performed to test differences in the number of significant intervals. Successive significant intervals were merged into single time windows and were reanalyzed with permutation procedures, and were plotted for easy reference.

## Results

### Behavioral results

#### Emotional facial assessments

**Fig. 2:**
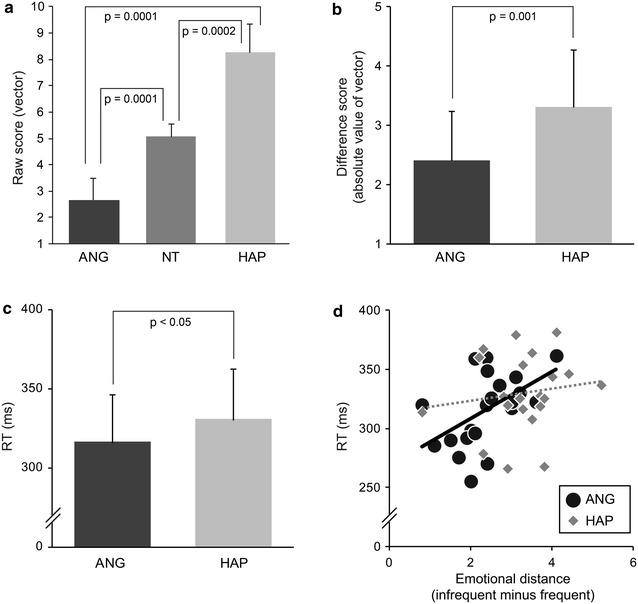
Behavioral results. **a** Emotional assessment scores of the angry (ANG), neutral (NT), and happy (HAP) faces by a ten-point Likert scale were compared by nonparametric tests. All pairs yielded significant differences. **b** Emotional distances (infrequent minus frequent) were directly compared between the ANG and HAP faces. The HAP faces were perceived to be more remote from the NT faces than the ANG faces. **c** Response times (RTs) for targets appearing immediately after infrequent stimuli were compared between the ANG and HAP faces. Responses to targets appearing after the HAP faces were relatively slower than those for targets appearing after the ANG faces and demonstrated an interference effect. *Error bars* in the graphs **a**–**c** represent standard deviations. **d** RTs were correlated with emotional distances for the ANG and HAP faces. RTs for the ANG faces significantly correlated with emotional distances (*r* = 0.516), while RTs for the HAP faces did not (*r* = 0.146)

Mean scores of emotional assessment were 5.0 ± 0.5 for the NT faces, 2.6 ± 0.8 for the ANG faces, and 8.2 ± 1.0 for the HAP faces. Scores of the three facial types were significantly different [Friedman: *χ*_(2)_^*2*^ = 40.095, *p* < 0.0001]. Further, pair-wise comparisons showed that all pairs were significantly different (Wilcoxon: NT vs. ANG, *Z* = 3.825, *p* = 0.0001; NT vs. HAP: *Z* = 3.785, *p* = 0.0002; ANG vs. HAP: *Z* = 3.825, *p* = 0.0001; Fig. 2a). Additionally, absolute emotional distances from the NT faces were compared between the ANG and HAP faces. The HAP faces were more remote from the NT faces than the ANG faces (HAP: 3.3 ± 0.9; ANG: 2.4 ± 0.8; *Z* = 3.312, *p* = 0.001; Fig. 2b).

#### Response times for targets

Mean RTs for targets immediately after three facial types were 325 ± 29 ms for the NT faces, 316 ± 31 ms for the ANG faces, and 330 ± 33 ms for the HAP faces. The RTs for targets after frequent NT and infrequent (ANG and HAP) faces were not significantly different [95 % CI (−1.964 to 2.056): NT vs. ANG, *t*_(20)_ = 1.132, *p* > 0.05; NT vs. HAP: *t*_(20)_ = 1.122, *p* > 0.05]. In contrast, RTs after the appearance of the HAP faces were significantly longer than those after the ANG faces [95 % CI (−1.964 to 2.056): *t*_(20)_ = 2.118, *p* < 0.05], suggesting an interference effect (Fig. 2c). To examine relational properties of intersubject variations of RTs in the ANG and HAP conditions, RTs were correlated with raw and different emotional assessment scores. The RTs for targets after the ANG faces positively correlated with emotional distances [95 % CI (−0.418 to 0.445): *r* = 0.516, *p* < 0.05, *df* = 19; Fig. 2d], which suggests that participants who perceived the ANG faces further than the NT faces responded relatively slower to targets with involuntary attention. On the other hand, the RTs after the HAP faces did not significantly correlate with emotional distances [95 % CI (−0.424 to 0.435): *r* = 0.146, *p* > 0.05, *df* = 19]. As observed in Fig. 2d, more than two-third of the participants (*n* = 15) judged that emotional distances between the NT and ANG faces were below the mean distance of 3 points. However, only seven participants (about 30 %) judged that distances between the NT and HAP faces were below 3 points. Accordingly, longer remote distances between the NT and HAP faces likely yielded relatively slower RTs (>300 ms) in many participants, which caused a smaller individual variation of RTs.

Impulsivity traits, on the other hand, did not significantly correlate with RTs for either the ANG faces [95 % CI (−0.435 to 0.438): AI, *r* = − 0.097, *p* > 0.05, *df* = 19; MI: *r* = − 0.132, *p* > 0.05, *df* = 19; NPI: *r* = − 0.20, *p* > 0.05, *df* = 19] or the HAP faces [95 % CI (−0.431 to 0.429): AI, *r* = − 0.114, *p* > 0.05, *df* = 19; MI: *r* = − 0.177, *p* > 0.05, *df* = 19; NPI: *r* = 0.129, *p* > 0.05, *df* = 19].

### Neurophysiological results

#### Fitting properties of frontal and occipital independent components

**Fig. 3:**
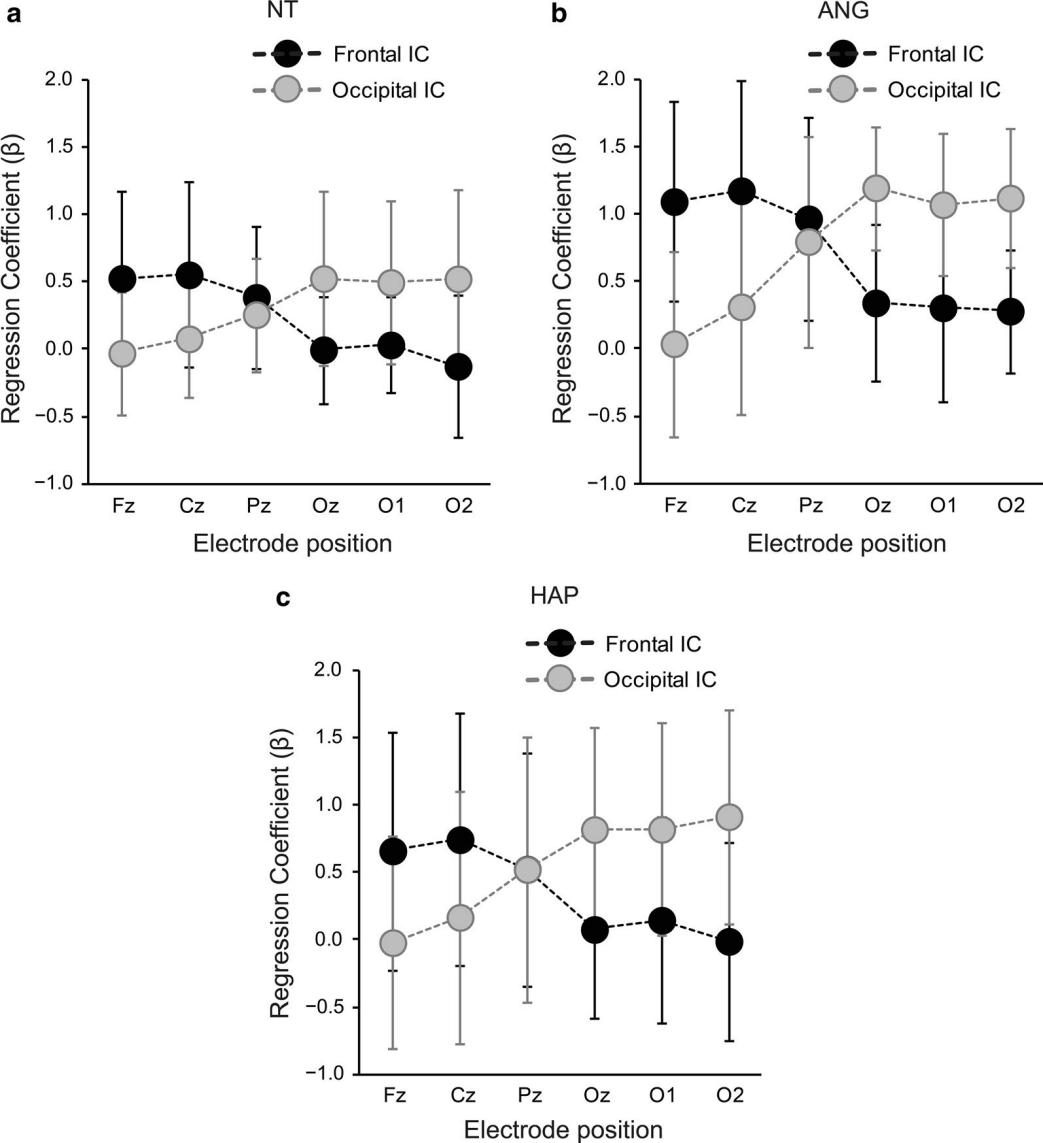
Fitting properties of frontal and occipital components in independent component analyses (ICA) to observed waveforms. Fitting properties were represented by regression coefficients (*β*) calculated by regression of average waveforms by ICs. The frontal IC yielded greater fitting properties (greater coefficients) in more anterior electrodes in the **a** neutral (NT), **b** angry (ANG), and **c** happy (HAP) faces. The occipital IC showed greater fitting properties in more posterior electrodes in all three facial conditions. *Error bars* represent standard deviations

Averaged ERP waveforms at the six electrodes in each condition were linearly regressed by the two ICs to obtain regression coefficients as an index of goodness-of-fit. As observed in Fig. 3a–c, the first component possessed greater coefficients in more anterior electrodes, and was defined as a frontal component. The second component yielded greater coefficients in more posterior electrodes (Oz, O1, and O2), and was defined as an occipital component. Coefficients of Fz and Oz were compared for each IC in each facial type. For all facial types, the first IC yielded greater coefficients for Fz than Oz (Wilcoxon: NT, *Z* = 2.624, *p* = 0.009; ANG: *Z* = 3.111, *p* = 0.002; HAP: *Z* = 2.240, *p* = 0.025). The second IC yielded greater coefficients for Oz than Fz (NT: *Z* = 2.416, *p* = 0.016; ANG: *Z* = 4.015, *p* < 0.0001; HAP: *Z* = 3.547, *p* = 0.0004). These results demonstrate that the present ICA successfully separated neural activities localized in frontal and occipital sites.

#### Amplitude comparisons between frequent and infrequent faces

**Fig. 4:**
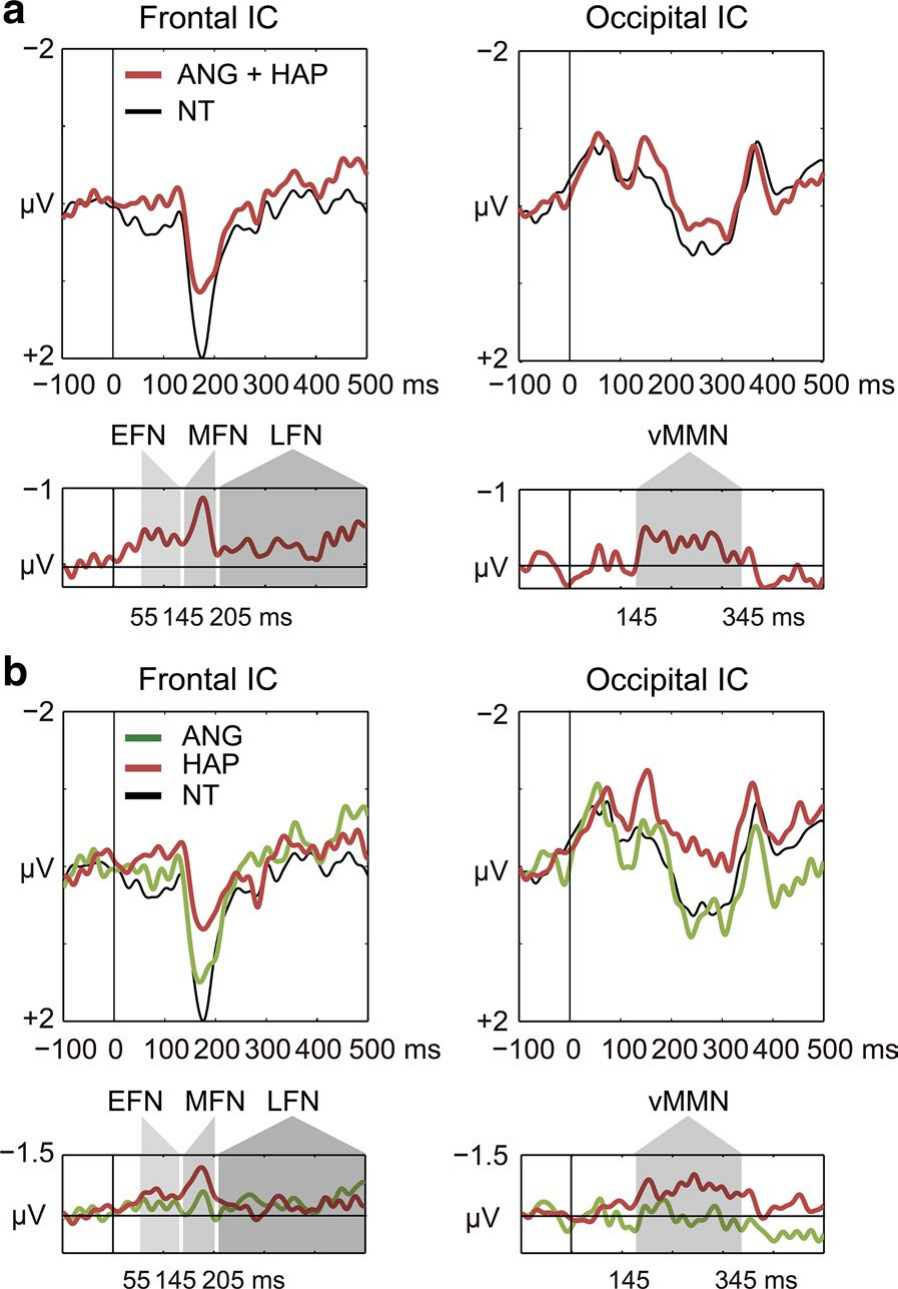
Comparisons of frontal and occipital independent components (ICs) between the frequent and infrequent conditions. **a** The infrequent condition combining the angry (ANG) and happy (HAP) faces was compared to the frequent neutral (NT) faces for the frontal (*left upper*) and occipital (*right upper*) ICs. Difference waveforms (infrequent minus frequent) are plotted in *lower portions*. Three phases of early frontal negativity (EFN), middle frontal negativity (MFN), and late frontal negativity (LFN) were morphologically specified for the frontal IC. The phase of the visual mismatch negativity (vMMN) was specified for the occipital IC. **b** The ANG and HAP faces were separately compared with the NT faces for the frontal (*left upper*) and occipital (*right upper*) ICs. Difference waveforms are plotted in* lower portions*. Three phases of the frontal IC and one phase of the occipital IC are shaded in* gray*

Based on visual inspection of the morphology of the difference waveforms (infrequent minus frequent) (left lower graph in Fig. 4a), three temporal phases of the frontal IC were defined, as described in the methodological section: the EFN phase (55–145 ms), the MFN phase (145–205 ms), and the LFN phase (205–500 ms). For the occipital IC (right lower graph in Fig. 4a), one temporal phase of a negative effect (145–345 ms) was defined as the vMMN phase.

Negative effects of the EFN, MFN, LFN, and vMMN for the combined infrequent condition (ANG and HAP) were not statistically significant in permutation paired *t* tests [95 % CI (−2.056 to 2.011): EFN, *t*_(20)_ = 1.798, *p* > 0.05; MFN: *t*_(20)_ = 1.829, *p* > 0.05; LFN: *t*_(20)_ = 1.486, *p* > 0.05; vMMN: *t*_(20)_ = 1.296, *p* > 0.05]. Accordingly, the infrequent condition was divided into the ANG and HAP conditions (Fig. 4b) and four temporal phases were tested by a one-way within-participants ANOVA with the emotion factor (NT, ANG, and HAP). For EFN, a main effect of emotion showed a significant trend [*F*_(2,40)_ = 2.543, *p* = 0.091, *η*_*p*_^*2*^ = 0.113] and hence, planned post hoc comparisons were performed to explore differences among the three facial types. The HAP faces yielded a significant effect compared to the NT faces (LSD: HAP vs. NT, *p* = 0.049; HAP vs. ANG: *p* = 0.194; NT vs. ANG: *p* = 0.333). For vMMN, a significant main effect of emotion was obtained in the ANOVA [*F*_(2,40)_ = 3.830, *p* = 0.030, *η*_*p*_^*2*^ = 0.161]. Post-hoc analyses demonstrated that the HAP, but not the ANG faces yielded a significant effect (HAP vs. NT: *p* = 0.035; HAP vs. ANG: *p* = 0.027; NT vs. ANG: *p* = 0.875). MFN showed a significant trend [*F*_(2,40)_ = 2.983, *p* = 0.062, *η*_*p*_^*2*^ = 0.130]. Planned pair-wise comparisons revealed that the HAP faces yielded a significant effect, compared to the NT faces (HAP vs. NT: *p* = 0.030; HAP vs. ANG: *p* = 0.115; NT vs. ANG: *p* = 0.547). LFN effects were not observed [*F*_(2,40)_ = 1.442, *p* = 0.248, *η*_*p*_^*2*^ = 0.067]. These results indicate that the HAP faces, compared to the ANG faces, yielded salient early and middle frontal negative and occipital vMMN effects for emotional change.

#### Temporally causal connection between early frontal and occipital vMMN effects

Intervals of EFN and vMMN were divided into sub-intervals with a 10 ms step and temporally constrained functional connections from EFN to vMMN were tested by permutation correlation analyses. To summarize main results, the HAP, but not ANG faces showed significant functional connectivity between EFN and vMMN for emotional change detection.

**Fig. 5:**
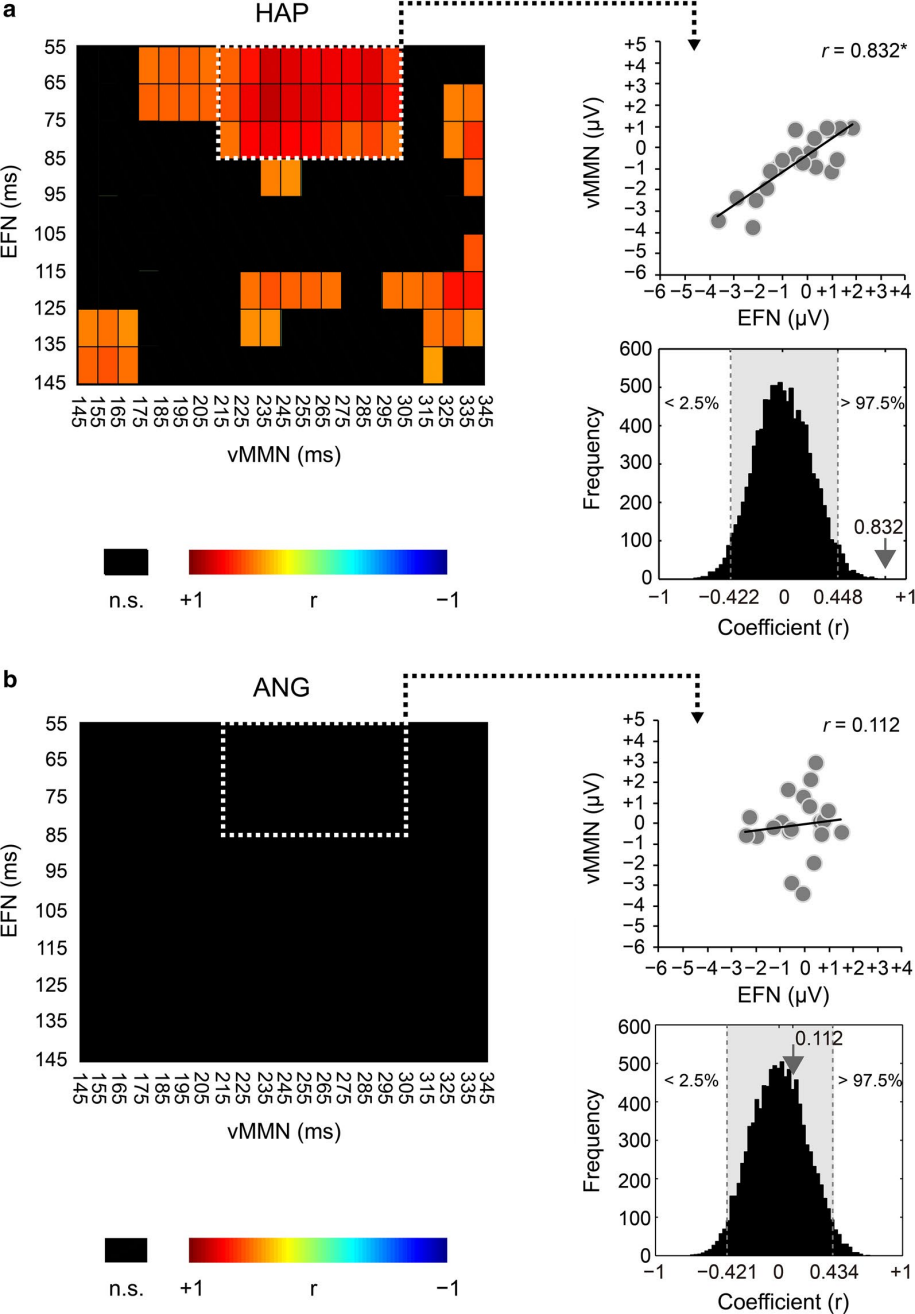
Correlations between early frontal negativity (EFN) and visual mismatch negativity (vMMN). **a** Correlation matrix (*left part*) for the happy (HAP) faces was filtered by an α level of *p* < 0.05. Gradual *red* *areas* represent intervals for significant correlations in permutation correlation analyses. Intervals with relatively strong correlations (3 EFN intervals × 9 vMMN intervals) are framed by a *white dotted square* and are summarized in a scatter diagram (*right upper*). Actual coefficient (*r* = 0.832) is outside the 95 % confidence interval (CI) (*gray area*) of 10,000 dummy coefficients (*right lower*). The *asterisk* indicates statistical significance of *p* < 0.05. **b** Correlation matrix (*left part*) for the angry (ANG) faces was filtered by an α level of *p* < 0.05. The ANG faces did not yield any significant interval. Intervals corresponding to the HAP faces are framed by a white dotted square and are summarized in a scatter diagram (*right upper*). Actual coefficient (*r* = 0.112) is within the 95 % CI (*gray area*) of the permutation distribution (*right lower*)

For relationships between EFN (9 intervals) and vMMN (20 intervals), 65 significant pairs were obtained among a total of 180 combinations for the HAP faces [mean *r* ± sd (range): 0.604 ± 0.119 (0.531–0.845), *p* < 0.05; Fig. 5a]. In particular, EFN in earlier intervals (3 intervals; 55–85 ms) strongly and positively correlated with vMMN (9 intervals; 215–305 ms) [*r* = 0.712 ± 0.10 (0.531–0.845), *p* < 0.05], which indicates that EFN enhanced subsequent occipital vMMN. The relation between merged EFN and vMMN was plotted for these time windows (*r* = 0.832, 95 % CI, −0.422 to 0.447, *p* < 0.05; Fig. 5a). For the ANG faces, on the other hand, none of the pairs were significantly different (range of *r*s: −0.295 to 0.380, *p* > 0.05; Fig. 5b). For comparison, the correlation between merged EFN (55–85 ms) and vMMN (215–305 ms) was plotted for these time windows (*r* = 0.112, 95 % CI, −0.421 to 0.434, *p* > 0.05; Fig. 5b). There is the apparent difference in the number of significant intervals between the HAP (65 pairs) and ANG (0) faces [emotion × significant interval: χ_(1)_^2^ = 76.900, *p* < 0.0001], which confirms the differences in fronto-occipital functional connectivity between the HAP and ANG faces.

#### Correlations between vMMN and behavioral measure

Each interval of EFN and vMMN were correlated with impulsivity traits (AI, MI, and NPI), RTs to targets, and emotional assessment scores (raw, difference) by a permutation procedure. Original coefficients and 95 % CIs of permutation distributions are summarized in the Additional file 1: Tables S1–S4. To overview main results, vMMNs for the HAP and ANG faces differently correlated with behavioral measures. vMMN for the HAP faces positively correlated with the MI sub-trait, while vMMN for the ANG faces positively correlated with RTs and emotional distances.

**Fig. 6:**
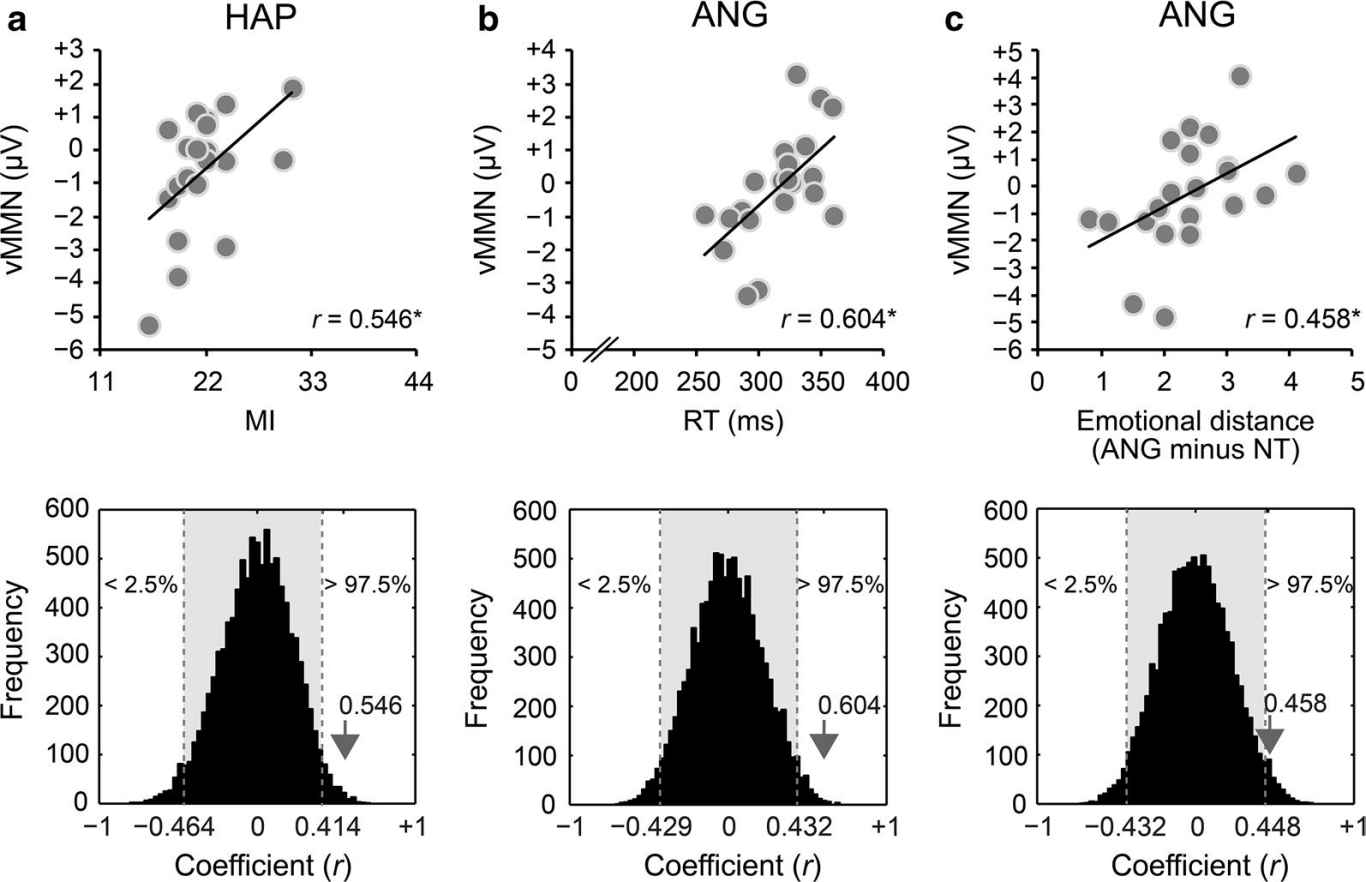
Correlations between visual mismatch negativity (vMMN) and behavioral measurements. **a** vMMN for the happy (HAP) faces positively correlated with the motor impulsivity (MI) trait (*r* = 0.546) (*upper graph*). The actual coefficient is outside the 95 % confidence interval (CI) of the permutation distribution (*lower graph*). The *asterisk* indicates statistical significance of *p* < 0.05. **b** vMMN for the angry (ANG) faces positively correlated with response time (RT) to targets (*upper graph*). The coefficient (*r* = 0.604) is observed outside the 95 % CI (*lower graph*). **c** vMMN for the ANG faces positively correlated with emotional distance [ANG minus neutral (NT)] (*upper graph*). The coefficient (*r* = 0.458) is also outside the 95 % CI (*lower graph*)

For impulsivity traits, vMMN for the HAP faces positively correlated with the MI in continuous intervals [155–225 ms: *r* = 0.535 ± 0.029 (0.491–0.559), *p* < 0.05; Additional file 1: Table S1], which is summarized in Fig. 6a (*r* = 0.546, 95 % CI, −0.464 to 0.414, *p* < 0.05). That is, higher or more negative vMMN for the HAP faces was associated with lower MI. vMMN for the ANG faces did not correlate with any sub-trait (Additional file 1: Table S2).

For RTs, vMMN for the HAP faces did not yield any significant correlation [*r* = − 0.085 ± 0.154 (−0.332 to 0.122), *p* > 0.05; Additional file 1: Table S3]. On the other hand, vMMN for the ANG faces positively correlated with RTs [155–225 ms: *r* = 0.542 ± 0.083 (0.437–0.636), *p* < 0.05; Additional file 1: Table S4]. Summary of merged results is represented in Fig. 6b (*r* = 0.604, 95 % CI, −0.429 to 0.432, *p* < 0.05), which demonstrates that greater vMMN for the ANG faces is related to faster RTs.

For emotional assessment, vMMN for the HAP faces did not significantly correlate with raw (HAP) and different (HAP minus NT) assessment scores [raw: *r* = 0.025 ± 0.042 (−0.081 to 0.123), *p* > 0.05; difference: *r* = − 0.273 ± 0.051 (−0.392 to 0.015), *p* > 0.05; Additional file 1: Table S3]. On the other hand, vMMN for the ANG faces positively correlated with emotional distances in restricted time windows (175–195 ms: e.g., 175–185 ms, *r* = 0.460, 95 % CI, −0.427 to 0.439, *p* < 0.05; Additional file 1: Table S4). Summary of merged data is represented in Fig. 6c (*r* = 0.458, 95 % CI, −0.432 to 0.448, *p* < 0.05), which reveals that greater vMMN for the ANG faces is associated with smaller emotional distances.

In contrast to vMMN, EFN did not robustly or continuously correlate with impulsivity and behavioral measures in either the HAP or ANG faces (Additional file 1: Tables S1–S4).

## Discussion

The present study conducted the neurophysiological experiment using a visual oddball paradigm to examine how impulsivity traits change emotional neural processing in healthy adults. To the best of our knowledge, this study is the first one to report neural modulation in detection of emotional face change by impulsivity in healthy populations. Based on the dual detector model, the early frontal negative and subsequent occipital vMMN effects likely corresponded to the transient detector mechanism and the stimulus-change detector mechanism, respectively, and hence, were mainly included into analyses. When we compared amplitudes between the infrequent and frequent faces, only the happy faces showed greater EFN and vMMN effects. These effects positively correlated with each other in a temporally constrained manner. An impulsivity sub-trait positively correlated only with vMMN for the happy faces, which indicates that impulsivity is associated selectively with vMMN, likely because of attenuated fronto-occipital functional connection for emotional change detection.

### Behavioral results

The participants showed a positive bias to the happy faces. They responded slower to targets immediately after the happy faces than those after the angry faces. This delayed RT effect for the happy faces is likely an interference effect produced by automatic arousal response [56, 57]. Arousal response to emotional stimuli can trigger an involuntary attention shift [58], even if attention is not intentionally directed to the stimuli [59]. All of participants reported after the experiment that they did not explicitly recognize emotional change and did not direct attention to the infrequent faces during the task. This also verifies the involuntary property of the interference effect in the present study.

On the other hand, emotional distances of the happy faces did not significantly correlate with the RTs, while distances of the angry faces positively correlated with the RTs. This discrepancy may result from dual properties of arousal response. Arousal is related not only to stimulus change, but also to stimulus significance [60]. For instance, meaningful stimuli, such as one´s own names as well as happy faces can disrupt inattentional blindness (namely, looking without seeing) [61, 62]. In the present study, the happy faces probably had not only more salient emotional properties, as has been observed in subjective emotional assessments, but also stimulus significance. The participants were probably affected by both these arousal factors and therefore, yielded uniform interference effects for the happy faces. Interestingly, target responses faster than the mean RT (about 300 ms) were observed for only three participants (14 %) for the happy faces, in contrast to eight participants (38 %) for the angry faces (Fig. 2d). Such positive emotional bias is likely related to endogenous emotional traits in the participants [56], because our experimental setting did not include factors evoking transient positive emotional states, such as joy [63] and feelings of, for instance, ‘Kawaii’ unique to Japanese popular culture [64]. The findings can be summarized as dual properties of arousal to the happy faces, which likely yielded relatively long RTs for the targets uniformly across the participants, and blocked a significant correlation between the RTs and emotional distances.

### Neurophysiological results

The present study conducted the ICA and separated the frontal and occipital components, which well fitted to averaged waveforms in the frontal and occipital electrodes, respectively, in goodness-of-fit regression tests. Although statistical independence of calculated components does not necessarily imply a unique source of neural populations [65], and the present study used the six electrodes not enough to estimate comprehensive neural sources, the localized frontal and occipital components may represent functionally segregated cortical activities.

Based on initial amplitude comparisons between the frequent and combined infrequent faces, frontal activities for emotional change were morphologically divided into three temporal phases of early, middle, and late negative deflections. On the other hand, occipital activities showed a main negative deflection during middle latencies. EFN and vMMN effects were significantly observed only for the happy faces when compared to neutral faces. Further, EFN and subsequent vMMN positively correlated with each other, which demonstrates that greater EFN enhanced vMMN in a temporally causal manner.

### Frontal negativity for infrequent happy faces

Consistent with the previous study reporting emotional modulation of N1 [66], the present study observed that EFN appeared for happy faces. Based on the dual detector model [22–24], EFN may be related to the transient detector mechanism. EFN (55–145 ms) appeared within the similar latency as N1 (75–125 ms) in the previous study [67], and hence, it likely corresponds to frontally-distributed visual N1. Because the participants were involuntarily affected by the happy faces, as represented by the interference effect (longer RTs) and emotional assessment after the experiments, EFN may be associated with automatic arousal to the happy faces.

On the other hand, this functional interpretation of EFN is seemingly inconsistent with previous findings. Vogel and Luck [67] observed that larger N1 effects appeared for more attentional target discrimination and suggested that N1 effects were not attributable to automatic arousal response. This difference between the previous and present findings may result from emotional properties of stimuli utilized. In contrast to Vogel and Luck’s experiment, which used alphabet stimuli, the present study applied emotional face stimuli. It has been widely acknowledged that emotional stimuli activate amygdala under both conscious and unconscious conditions [68]. Direct subcortical pathways from amygdala to cortical areas are likely fundamental for the early emotional sensory processing [69, 70]. Happy faces in the present study may increase amygdala activations for un-masked emotional processing [68] during early latencies (<140 ms post-stimulus). Early amygdala activity promotes neural activities in connected regions in the prefrontal cortex (PFC), such as ventrolateral PFC (e.g., BA10) and the rostral anterior cingulate cortex [71] for autonomic arousal response [72]. EFN in the present study may be related to such neural activities in subcortical pathways for the automatic sensory processing of emotional stimuli.

Another concern is the polarity of the early frontal effect in the present study. Frontal effects for stimulus change have been observed in previous studies [9, 10, 45, 73, 74]. Astikainen et al. [45] argues that negative polarity of frontal effects induces the involuntary attentional shift to deviant stimuli. Wei et al. [75] reported the supportive finding that pre-attentive processing of visual stimuli with deviant contrast yielded more negative frontal effects around 140 ms post-stimulus than attentional processing. Conversely, frontal positivity effects have been inconsistently reported in several vMMN experiments (non-emotional: [9, 45]; emotional: [10, 76, 77]). Eimer and Holmes [76] observed early frontal positive effects (110–150 ms) before face identification and argued that the frontal positivity was associated with an automatic detection of facial emotions before conscious identification. Another study argues that frontal positive effects are associated with refractoriness to frequent stimuli in a condition where stimulus deviancy is remarkable, as in an antagonistic color condition [9]. Astikainen and Hietanen [10] also observed frontal positive effects to deviant faces around 150 ms, in addition to vMMN, and suggested several possible interpretations, including that frontal positive effects reflect involuntary direction of attention to deviant emotional faces (see also, [76]). The automatic property of the early frontal positivity is also supported by another study reporting subliminal frontal positivity (140–180 ms) for emotional facial processing [78].

**Fig. 7:**
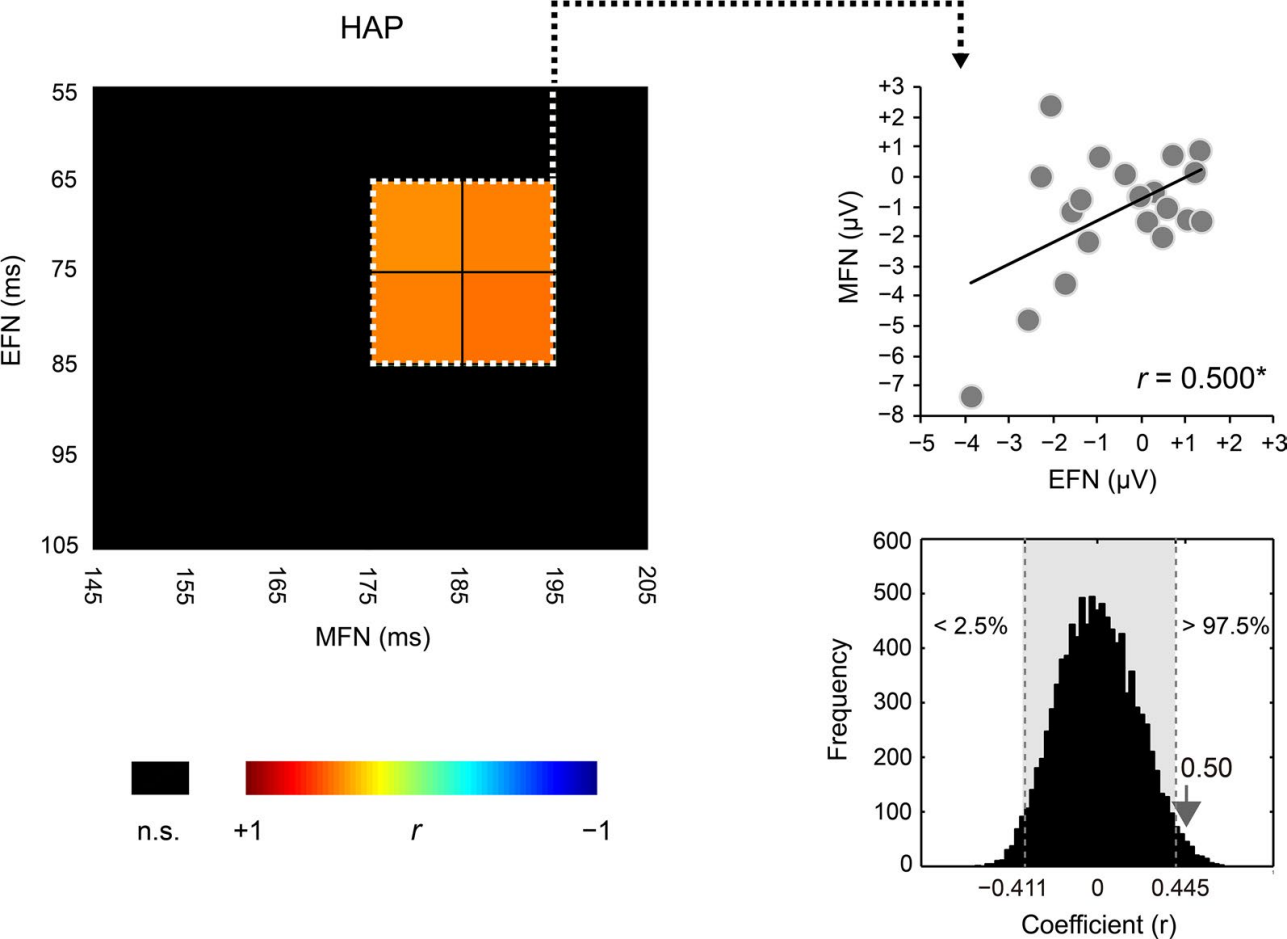
Correlation between early frontal negativity (EFN) and middle frontal negativity (MFN) for the happy (HAP) faces. Correlation matrix (*left part*) was filtered by an α level of *p* < 0.05. Gradual *red areas* represent intervals for significant correlations in permutation correlation analyses. EFN (65–85 ms) positively correlated with MFN (175–195 ms) (see for summary, a *right upper scatter diagram*). Actual coefficient (*r* = 0.500) is outside the 95 % confidence interval (*gray area*) of 10,000 dummy coefficients (a *right lower* histogram). The *asterisk* indicates statistical significance of *p* < 0.05

Despite previous competitive findings, the present behavioral results argue that the EFN for the happy faces is associated with automatic arousal to emotional change, and the subsequent MFN with involuntary attentional shift. In this study, EFN was observed earlier than it was in previous studies (100–200 ms). This suggests that EFN reflects more basic and earlier neurophysiological response than previous frontal effects and automatic arousal triggering involuntary attentional shift [58]. MFN (approximately 180 ms) is similar to those of previous frontal effects. Hence, the MFN probably corresponds to previous frontal effects related to the involuntary attentional shift triggered by EFN. Interestingly, EFN (65–85 ms) positively correlates with MFN (175–195 ms) in a temporally causal manner (*r* = 0.500, 95 % CI, −0.411 to 0.445, *p* < 0.05; Fig. 7), which supports the argument that EFN for automatic arousal promotes MFN for the involuntary attentional shift.

In summary, that basic arousal response to emotional change first evokes an early frontal negativity and subsequently a middle frontal negativity, which may correspond to a previous frontal negative effect. If the early frontal negativity does not appear, frontal positivity may appear alternatively in latencies similar with those of MFN, as shown in previous studies. Future studies are required for precise examination of background neural and functional mechanisms.

### Occipital vMMN for infrequent happy faces

The present study also observed negative potential effects in occipital areas only for the happy faces. Because these posterior dominant negative effects appeared in the latency from about 100 to 300 ms post-stimulus, it is likely vMMN, as has been observed in previous studies [10, 40]. This result also supports the present behavioral findings that the happy faces evoked salient effects of emotional change in the participants, and yielded interference effects for target response. Based on the dual detector model, MMN is related to the stimulus-change detector mechanism for a short-term sensory memory trace of preceding stimuli [20]. Because the happy faces were more salient than the angry faces for the present participants, the happy faces were likely more dissociated from a short-term memory representation of neutral faces and consequently yielded vMMN.

vMMN for the happy faces had temporally causal connection with EFN, which provides meaningful information about early phases of emotional facial processing. The happy faces, compared to the angry faces, yielded an interference effect for the target response and likely consumed more attentional resources. During emotional facial processing under more attentive conditions, arousal neural activities may trigger later mismatch neural activities through a fronto-occipital functional connection until about 300 ms post-stimulus. The fronto-occipital connection is defined as a feedback connection between prefrontal and sensory areas. Although the prefrontal sources of EFN are not clear in the present study, the prefrontal areas can initiate activation within 100 ms after visual inputs through feed forward pathways from the sensory or subcortical areas including the amygdala to the higher cortical areas [57, 79]. Such automatic prefrontal activities are fast enough to modify neural activations in the visual areas through feedback pathways and likely affect vMMN elicitation.

### Correlation between vMMN for the infrequent happy faces and impulsivity traits

The impulsive sub-trait of motor impulsivity correlated with vMMN, but not with EFN for the happy faces. This implies that impulsivity is related to weakened fronto-occipital functional connection for emotional facial processing and selectively affects vMMN. In healthy people with normal impulsivity, automatic arousal response to salient emotional change may be evoked in general. However they may be affected sensitively by impulsivity in later stimulus-change detection, showing the correlation only between impulsivity and vMMN. It is unclear whether people with pathological impulsivity show similar relational patterns under the present experimental settings. A previous study suggests that there are pathological gaps between healthy and clinical populations and they yield different patterns in correlations between impulsivity and MMN. Hung et al. [80] used an auditory mismatch paradigm and examined MMN effects in twenty younger delinquents with a history of severe aggressive behaviors. They observed that MMN to fearful stimuli was negatively correlated with impulsivity: that is, higher impulsivity was associated with greater, more negative MMN in a reverse manner. On the contrary to healthy populations, certain populations with abnormal impulsivity may respond more selectively to emotional saliency that is potentially related to abnormal behaviors and enhance sensori-perceptual change detection.

Correlational properties of the angry faces also emphasize impulsivity-relevant modulation in processing of the happy faces. EFN and vMMN for the angry faces did not significantly correlate with each other and with impulsivity traits. However, vMMN for the angry faces positively correlated with the RTs for the target response and emotional distances from the frequent neutral faces, which indicated that smaller or more positive vMMNs were related to longer RTs and larger emotional distances. This suggests that changes in memory-based detector processing reflected by vMMN are differently related to impulsivity traits and emotional properties of sensory inputs. The angry faces in the present study yielded larger individual differences in emotional assessment, which were positively correlated with the RTs. Participants who answered smaller emotional distances between the angry and neutral faces might use more neural resources for mismatched face processing and yield larger vMMN. On the other hand, only one participant assessed emotional distances between the happy and neutral faces below two points, in contrast to seven participants for the angry faces. Taken into consideration this difference between the happy and angry conditions, when emotional distances are large enough and relatively uniform across individuals, modulation of vMMN by impulsivity may manifest itself, as observed in the present happy face condition.

To summarize, healthy people normally evoke the automatic arousal response to salient emotional change, but individuals with higher impulsivity attenuate vMMN for emotional change detection, likely because of the weakened fronto-occipital feedback functional connection.

The present study possess several limitations that should be addressed for future studies. First, we did not include a control condition, where neutral, angry, and happy faces were presented in equal proportions. This prevents us from precisely answering the question of whether the vMMN for the happy face in the present study reflects detection of emotional regularity violation. When major concerns of future studies are not only emotional change, but also emotional regularity violation, we should also prepare a control condition compared with a deviant condition.

Second, a relatively small number of the infrequent stimuli and the electrodes were used in the present study. We should re-consider a number of stimuli and electrodes usable without excess burden on, in particular, clinical populations for future studies.

## Conclusions

The present study revealed that our healthy participants showed positive bias to happy faces and yielded vMMN effects that were causally connected with early frontal negativity. However, this functional connection was likely affected by impulsivity traits and only vMMN correlated with impulsivity traits. This suggests that the early frontal negativity for the automatic arousal to emotional change can occur, but this frontal activity does not always effectively trigger subsequent mismatch neural activities in a correlated manner, at least, for healthy people with higher but not pathological impulsivity. These findings imply that if vMMN for salient emotional change is strongly attenuated or does not appear in specific populations, it may link to current or future symptoms related to pathological impulsivity. Furthermore, if EFN shows similar patterns of abnormal activities, there may be potential defects in sensori-perceptual monitoring of meaningful information for our lives. Early frontal and middle occipital mismatch neural activities might function as markers used to prevent developing pathological states.

## Additional file

10.1186/s12868-015-0223-x Summary of permutation correlation analyses between neurophysiological (early frontal and subsequent occipital ERP effects) and behavioral (impulsivity traits, emotional assessments, and task performances) measures.
