# Supplementary material for: Neurophysiological modulation of rapid emotional face processing is associated with impulsivity traits
Source: BMC Neurosci. 2015 Dec 9;16:87. doi: 10.1186/s12868-015-0223-x (PMC4675059; doi:10.1186/s12868-015-0223-x)
Supplement: Supplementary file 1 — 10.1186/s12868-015-0223-x Summary of permutation correlation analyses between neurophysiological (early frontal and subsequent occipital ERP effects) and behavioral (impulsivity traits, emotional assessments, and task performances) measures. [file 12868_2015_223_MOESM1_ESM.pdf]

S1 Table. Results of permutation correlation analyses (10000 re-sampling for each analysis) for event-related potentials (ERPs) and impulsivity traits (BIS−11) for the happy faces (*n* = 21).

ERPs	Interval (ms)	AI			MI			NPI		
		r	95% CI low	95% CI high	*r*	95% CI low	95% CI high	*r*	95% CI low	95% CI high
EFN	55–65	0.066	−0.434	0.437	0.045	−0.435	0.434	−0.127	−0.428	0.433
	65–75	0.165	−0.438	0.445	0.093	−0.449	0.427	−0.179	−0.432	0.439
	75–85	−0.001	−0.433	0.438	−0.032	−0.448	0.417	−0.345	−0.431	0.426
	85–95	−0.268	−0.434	0.442	−0.378	−0.428	0.433	**−0.466***	−0.424	0.432
	95–105	−0.186	−0.426	0.431	−0.245	−0.439	0.416	−0.414	−0.419	0.456
	105–115	0.074	−0.430	0.428	0.047	−0.441	0.427	−0.289	−0.428	0.436
	115–125	0.201	−0.427	0.443	0.082	−0.444	0.421	−0.071	−0.425	0.437
	125–135	0.204	−0.436	0.429	0.235	−0.428	0.427	0.061	−0.433	0.427
	135–145	0.203	−0.438	0.434	0.324	−0.410	0.459	0.107	−0.445	0.431
vMMN	145–155	−0.018	−0.434	0.432	0.350	−0.443	0.419	−0.018	−0.430	0.436
	155–165	0.146	−0.433	0.431	**0.491***	−0.450	0.417	0.072	−0.432	0.440
	165–175	0.211	−0.426	0.434	**0.555***	−0.443	0.419	0.104	−0.420	0.444
	175–185	0.343	−0.429	0.441	**0.556***	−0.440	0.418	0.113	−0.421	0.445
	185–195	0.422	−0.425	0.429	**0.547***	−0.442	0.417	0.118	−0.422	0.448
	195–205	0.387	−0.414	0.449	**0.543***	−0.447	0.411	0.121	−0.417	0.442
	205–215	0.325	−0.422	0.456	**0.559***	−0.454	0.412	0.123	−0.419	0.447
	215–225	0.239	−0.425	0.438	**0.494***	−0.453	0.420	0.045	−0.424	0.441
	225–235	0.102	−0.427	0.446	0.279	−0.457	0.424	−0.061	−0.426	0.436
	235–245	0.039	−0.420	0.429	0.181	−0.441	0.426	−0.028	−0.429	0.424
	245–255	0.059	−0.426	0.429	0.207	−0.442	0.424	−0.028	−0.431	0.446
	255–265	0.164	−0.423	0.439	0.302	−0.442	0.425	−0.022	−0.429	0.434
	265–275	0.313	−0.430	0.433	0.366	−0.441	0.422	0.138	−0.429	0.438
	275–285	0.391	−0.431	0.429	0.384	−0.439	0.429	0.192	−0.431	0.441
	285–295	0.364	−0.435	0.440	0.362	−0.443	0.424	0.104	−0.436	0.447
	295–305	0.330	−0.427	0.434	0.251	−0.438	0.416	0.084	−0.418	0.443
	305–315	0.171	−0.433	0.441	0.210	−0.439	0.432	0.014	−0.418	0.445
	315–325	0.048	−0.424	0.439	0.207	−0.456	0.415	−0.065	−0.424	0.440
	325–335	−0.026	−0.416	0.438	−0.003	−0.450	0.420	−0.162	−0.418	0.444
	335–345	−0.167	−0.412	0.441	−0.099	−0.458	0.422	−0.258	−0.433	0.447

*AI: attentional impulsivity; MI: motor impulsivity; NPI: non-planning impulsivity; EFN: early frontal negativity; vMMN: visual mismatch negativity; CI: confidence interval; *p < 0.05*

S2 Table. Results of permutation correlation analyses (10000 re-sampling for each analysis) for event-related potentials (ERPs) and impulsivity traits (BIS−11) for the angry faces (*n* = 21).

ERPs	Interval (ms)	AI			MI			NPI		
		r	95% CI		*r*	95% CI		*r*	95% CI	
			low	high		low	high		low	high
EFN	55–65	0.192	−0.435	0.431	0.405	−0.429	0.428	0.101	−0.430	0.438
	65–75	0.215	−0.429	0.433	0.278	−0.437	0.420	0.090	−0.424	0.437
	75–85	0.094	−0.426	0.439	0.157	−0.442	0.418	−0.102	−0.427	0.444
	85–95	−0.086	−0.426	0.439	−0.034	−0.431	0.426	−0.242	−0.429	0.437
	95–105	−0.007	−0.426	0.439	0.013	−0.438	0.432	−0.393	−0.430	0.437
	105–115	0.174	−0.430	0.424	0.176	−0.434	0.432	−0.411	−0.436	0.429
	115–125	0.058	−0.428	0.440	0.076	−0.459	0.419	−0.158	−0.425	0.449
	125–135	−0.197	−0.432	0.432	0.103	−0.427	0.431	0.095	−0.438	0.429
	135–145	−0.176	−0.444	0.398	0.323	−0.403	0.496	−0.003	−0.470	0.412
vMMN	145–155	0.111	−0.425	0.427	−0.109	−0.441	0.424	−0.083	−0.414	0.435
	155–165	0.249	−0.443	0.424	0.143	−0.429	0.440	−0.016	−0.448	0.425
	165–175	0.362	−0.431	0.433	0.290	−0.429	0.444	0.043	−0.441	0.434
	175–185	0.370	−0.421	0.437	0.333	−0.443	0.422	0.090	−0.430	0.436
	185–195	0.334	−0.440	0.433	0.389	−0.444	0.430	0.199	−0.430	0.437
	195–205	0.316	−0.424	0.430	0.388	−0.447	0.423	0.238	−0.422	0.445
	205–215	0.327	−0.421	0.446	0.421	−0.457	0.422	0.158	−0.414	0.455
	215–225	0.265	−0.434	0.435	0.327	−0.449	0.432	0.113	−0.428	0.440
	225–235	0.247	−0.434	0.424	0.165	−0.429	0.443	0.188	−0.446	0.427
	235–245	0.306	−0.442	0.419	0.155	−0.418	0.451	0.287	−0.433	0.417
	245–255	0.207	−0.441	0.431	0.030	−0.418	0.445	0.298	−0.447	0.426
	255–265	0.181	−0.438	0.425	−0.046	−0.429	0.442	0.221	−0.439	0.417
	265–275	0.283	−0.433	0.436	0.048	−0.430	0.428	0.084	−0.434	0.418
	275–285	0.283	−0.425	0.427	0.090	−0.424	0.432	0.092	−0.436	0.431
	285–295	0.204	−0.430	0.427	0.112	−0.435	0.435	0.079	−0.436	0.443
	295–305	0.124	−0.429	0.438	0.026	−0.434	0.438	−0.040	−0.426	0.440
	305–315	−0.033	−0.429	0.438	−0.059	−0.457	0.420	−0.204	−0.427	0.449
	315–325	−0.137	−0.441	0.431	−0.022	−0.419	0.440	−0.324	−0.444	0.430
	325–335	−0.183	−0.436	0.425	−0.215	−0.434	0.431	−0.370	−0.427	0.427
	335–345	−0.177	−0.429	0.437	−0.333	−0.425	0.435	−0.329	−0.423	0.434

AI: attentional impulsivity; MI: motor impulsivity; NPI: non-planning impulsivity; EFN: early frontal negativity; vMMN: visual mismatch negativity; CI: confidence interval

S3 Table. Results of permutation correlation analyses (10000 re-sampling for each analysis) for event-related potentials (ERPs) and behavioral measure (response time, emotional assessment) for the happy faces (*n* = 21).

ERPs	Interval (ms)	RT			Emotional assessment			Emotional distance		
		r	95% CI low	95% CI high	*r*	95% CI low	95% CI high	*r*	95% CI low	95% CI high
EFN	55–65	0.019	−0.425	0.434	0.164	−0.432	0.432	−0.197	−0.416	0.431
	65–75	−0.032	−0.443	0.438	0.132	−0.444	0.430	−0.224	−0.404	0.459
	75–85	−0.115	−0.427	0.444	0.208	−0.439	0.433	−0.343	−0.396	0.451
	85–95	−0.150	−0.435	0.435	0.315	−0.440	0.431	−0.313	−0.420	0.438
	95–105	−0.152	−0.423	0.438	0.377	−0.453	0.414	−0.267	−0.389	0.493
	105–115	−0.096	−0.422	0.441	0.334	−0.440	0.420	−0.235	−0.417	0.478
	115–125	−0.063	−0.428	0.442	0.201	−0.456	0.412	−0.158	−0.383	0.466
	125–135	−0.276	−0.428	0.439	0.315	−0.419	0.431	−0.293	−0.413	0.421
	135–145	−0.258	−0.444	0.436	0.289	−0.423	0.454	−0.286	−0.465	0.387
vMMN	145–155	−0.263	−0.429	0.432	−0.040	−0.436	0.430	−0.277	−0.443	0.471
	155–165	−0.332	−0.427	0.444	0.071	−0.451	0.422	−0.319	−0.413	0.503
	165–175	−0.260	−0.427	0.452	0.071	−0.447	0.422	−0.323	−0.419	0.499
	175–185	−0.147	−0.427	0.440	0.028	−0.448	0.410	−0.261	−0.388	0.477
	185–195	−0.208	−0.425	0.428	0.005	−0.447	0.430	−0.183	−0.391	0.485
	195–205	−0.296	−0.419	0.444	−0.051	−0.460	0.408	−0.127	−0.377	0.521
	205–215	−0.306	−0.416	0.438	−0.063	−0.459	0.413	−0.146	−0.386	0.508
	215–225	−0.221	−0.427	0.452	0.016	−0.440	0.408	−0.276	−0.373	0.505
	225–235	−0.079	−0.425	0.445	0.112	−0.453	0.414	−0.383	−0.397	0.484
	235–245	0.000	−0.437	0.432	0.096	−0.444	0.421	−0.384	−0.406	0.458
	245–255	0.069	−0.422	0.437	0.042	−0.438	0.415	−0.390	−0.402	0.449
	255–265	0.072	−0.428	0.443	0.095	−0.443	0.416	−0.392	−0.405	0.458
	265–275	0.097	−0.432	0.442	0.063	−0.440	0.423	−0.323	−0.423	0.451
	275–285	0.122	−0.436	0.438	−0.075	−0.442	0.421	−0.211	−0.430	0.443
	285–295	−0.016	−0.430	0.436	−0.081	−0.449	0.429	−0.069	−0.417	0.462
	295–305	−0.001	−0.427	0.438	−0.031	−0.445	0.420	−0.015	−0.405	0.485
	305–315	0.098	−0.423	0.436	−0.001	−0.449	0.425	−0.137	−0.385	0.479
	315–325	0.000	−0.428	0.445	0.086	−0.438	0.418	−0.227	−0.392	0.479
	325–335	−0.010	−0.425	0.447	0.183	−0.460	0.415	−0.286	−0.384	0.512
	335–345	−0.013	−0.414	0.443	0.172	−0.452	0.420	−0.359	−0.410	0.533

RT: response time; EFN: early frontal negativity; vMMN: visual mismatch negativity; CI: confidence interval

S4 Table. Results of permutation correlation analyses (10000 re-sampling for each analysis) for event-related potentials (ERPs) and behavioral measure (response time, emotional assessment) for the angry faces (*n* = 21).

ERPs	Interval (ms)	RT			Emotional assessment			Emotional distance		
		r	95% CI low	95% CI high	*r*	95% CI low	95% CI high	*r*	95% CI low	95% CI high
EFN	55–65	−0.133	−0.431	0.437	−0.127	−0.444	0.433	−0.070	−0.429	0.431
	65–75	−0.088	−0.441	0.430	−0.235	−0.443	0.417	0.019	−0.404	0.446
	75–85	−0.109	−0.426	0.438	−0.229	−0.441	0.429	−0.018	−0.402	0.458
	85–95	−0.081	−0.429	0.435	−0.122	−0.443	0.420	−0.215	−0.426	0.446
	95–105	−0.023	−0.432	0.429	−0.001	−0.426	0.433	−0.343	−0.429	0.430
	105–115	−0.029	−0.423	0.430	0.124	−0.441	0.424	−0.378	−0.426	0.451
	115–125	−0.004	−0.432	0.440	0.280	−0.454	0.425	−0.291	−0.415	0.457
	125–135	0.024	−0.432	0.431	0.380	−0.431	0.437	−0.130	−0.446	0.437
	135–145	0.003	−0.434	0.415	0.327	−0.400	0.463	−0.163	−0.504	0.384
vMMN	145–155	**0.481***	−0.425	0.440	−0.088	−0.436	0.426	0.045	−0.419	0.457
	155–165	**0.582***	−0.447	0.429	0.086	−0.422	0.454	0.293	−0.464	0.420
	165–175	**0.636***	−0.429	0.421	0.142	−0.430	0.442	0.407	−0.443	0.426
	175–185	**0.625***	−0.430	0.436	0.225	−0.438	0.439	**0.460***	−0.427	0.439
	185–195	**0.492***	−0.432	0.434	0.251	−0.430	0.430	**0.446***	−0.443	0.446
	195–205	0.396	−0.421	0.437	0.235	−0.448	0.418	0.394	−0.414	0.451
	205–215	0.418	−0.426	0.442	0.306	−0.447	0.410	0.414	−0.413	0.465
	215–225	**0.437***	−0.438	0.436	0.345	−0.436	0.436	0.417	−0.428	0.440
	225–235	0.312	−0.438	0.431	0.284	−0.422	0.446	0.373	−0.455	0.428
	235–245	0.076	−0.436	0.419	0.075	−0.408	0.456	0.237	−0.465	0.401
	245–255	0.004	−0.436	0.434	−0.136	−0.418	0.450	0.070	−0.456	0.406
	255–265	0.110	−0.435	0.429	−0.061	−0.420	0.450	0.154	−0.445	0.424
	265–275	0.233	−0.443	0.438	0.118	−0.422	0.432	0.248	−0.433	0.431
	275–285	0.277	−0.426	0.432	0.107	−0.432	0.433	0.131	−0.446	0.434
	285–295	0.278	−0.432	0.443	0.093	−0.440	0.433	0.120	−0.440	0.441
	295–305	0.240	−0.427	0.437	0.040	−0.441	0.444	0.098	−0.424	0.442
	305–315	0.034	−0.426	0.436	−0.101	−0.437	0.427	−0.154	−0.422	0.448
	315–325	−0.090	−0.442	0.437	−0.077	−0.431	0.446	−0.217	−0.437	0.416
	325–335	−0.007	−0.428	0.426	−0.016	−0.435	0.435	−0.081	−0.421	0.435
	335–345	0.067	−0.437	0.421	−0.050	−0.427	0.437	−0.070	−0.450	0.427

RT: response time; EFN: early frontal negativity; vMMN: visual mismatch negativity; CI: confidence interval; *$p < 0.05$
